# A Heuristic Model of Alcohol Dependence

**DOI:** 10.1371/journal.pone.0092221

**Published:** 2014-03-21

**Authors:** Zhen Qi, Felix Tretter, Eberhard O. Voit

**Affiliations:** 1 Department of Biomedical Engineering, Georgia Institute of Technology and Emory University Medical School, Atlanta, Georgia, United States of America; 2 Integrative BioSystems Institute, Georgia Institute of Technology, Atlanta, Georgia, United States of America; 3 Center for Neurodegenerative Disease, Emory University School of Medicine, Atlanta, Georgia, United States of America; 4 Isar-Amper-Klinikum gemeinnützige GmbH, Klinikum München-Ost, Haar, Landkreis München, Germany; Universidad de La Laguna, Spain

## Abstract

**Background:**

Substance dependence poses a critical health problem. Sadly, its neurobiological mechanisms are still unclear, and this lack of real understanding is reflected in insufficient treatment options. It has been hypothesized that alcohol effects are due to an imbalance between neuroexcitatory and neuroinhibitory amino acids. However, glutamate and GABA interact with other neurotransmitters, which form a complicated network whose functioning evades intuition and should be investigated systemically with methods of biomedical systems analysis.

**Methods and Results:**

We present a heuristic model of neurotransmitters that combines a neurochemical interaction matrix at the biochemical level with a mobile describing the balances between pairs of neurotransmitters at the physiological and behavioral level. We investigate the effects of alcohol on the integrated neurotransmitter systems at both levels. The model simulation results are consistent with clinical and experimental observations. The model demonstrates that the drug diazepam for symptoms of alcohol withdrawal effectively reduces the imbalances between neurotransmitters. Moreover, the acetylcholine signal is suggested as a novel target for treatment of symptoms associated with alcohol withdrawal.

**Conclusions:**

Efficient means of integrating clinical symptoms across multiple levels are still scarce and difficult to establish. We present a heuristic model of systemic neurotransmitter functionality that permits the assessment of genetic, biochemical, and pharmacological perturbations. The model can serve as a tool to represent clinical and biological observations and explore various scenarios associated with alcohol dependence and its treatments. It also is very well suited for educational purposes.

## Introduction

“Substance dependence” describes a physiological and psychological state in which a user has developed a dependence on such drugs as alcohol, tobacco, opiates or amphetamines. Substance dependence is nothing new, as alcohol use and abuse were already observed in ancient Egypt and Greece [1], [2]. The *Diagnostic and Statistical Manual of Mental Disorders* [3] defines: “When an individual persists in use of alcohol or other drugs despite problems related to use of the substance, substance dependence may be diagnosed. Compulsive and repetitive use may result in tolerance to the effect of the drug and withdrawal symptoms when use is reduced or stopped.” Substance dependence is unfortunately quite prevalent. The lifetime prevalence of alcohol dependence was reported to be 14% in a non-institutionalized adult population (ages: 15–54 years) [3], and alcohol abuse is associated with 85,000 deaths annually in the United States alone [4]. In 2000, there were over 1.2 billion smokers worldwide [5], and it is estimated that there are 24.8 million amphetamine users worldwide [6].

**Figure 1:**
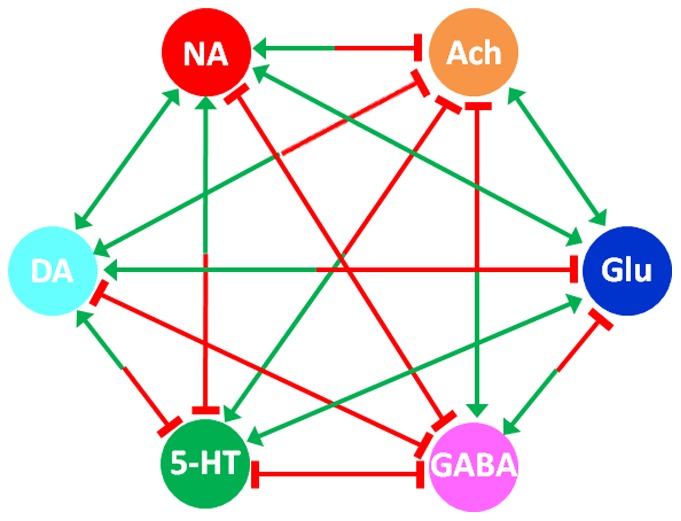
The neurochemical interaction matrix. The interactions among six neurotransmitter systems in the human brain form a fully connected network, which may be represented as a matrix. Arrows represent activation; while bar-headed lines represent inhibition. Abbreviation: dopamine (DA), acetylcholine (ACh), serotonin (5-HT), glutamate (Glu), noradrenaline (NA), and gamma-aminobutyric acid (GABA).

Depending on the type of substance dependence, different neurotransmitters have been suggested as targets. Specifically, alcohol can increase inhibitory neurotransmission (*e.g.*, through gamma-aminobutyric acid (GABA)), while simultaneously reducing excitatory neurotransmission (*e.g.*, through glutamate (Glu)) [7]. Substances like amphetamine increase the dopamine level in the synaptic cleft [8], whereas nicotine mimics psychopharmacological effects of the neurotransmitter acetylcholine and modulates dopamine release [9], [10]. In addition to modulation of direct targets, addictive substances can indirectly modulate other neurotransmitters through neuronal projections and metabolic pathways. For example, alcohol can directly reduce glutamate level in the *striatum*, which in turn regulates GABAergic activity in the *nucleus accumbens*. As a consequence, alcohol can have an indirect effect on the GABA level in the *nucleus accumbens*. In this manner, all neurotransmitters interact with each other directly or indirectly, as it is shown in Figure 1. These interactions imply that behavioral effects of addictive substances are not merely associated with direct targets. Instead, both direct and indirect effects should be investigated in an integrated fashion involving all neurotransmitter systems. This conclusion is in line with clinical experience showing that altered function of any individual neurotransmitter alone cannot explain a complex syndrome such as the development of alcoholism, withdrawal craving and relapse.

Alcohol withdrawal syndrome is one of the criteria for alcohol dependence according to DSM-IV [3]. It follows alcohol abuse and exhibits symptoms of autonomic hyperactivity, tremor, anxiety and restlessness. Some individuals also have seizures, hallucinations and, possibly, delirium. As a common medical problem, alcohol withdrawal syndrome has had a mortality as high as 15% in the past [11], which later improved to 2% due to better recognition and treatment [12].

Studies have shown that alcohol withdrawal is associated with an increase in glutamate in the *striatum* [13], the *nucleus accumbens* [14], the *hippocampus* [15], and the cerebrospinal fluid [16]. Changes in the inhibitory activity of GABA are also observed and connected to hyper-excitability [17]. Since alcohol can affect neurotransmitters in the brain and modify behaviors, a general perturbation of the brain’s neurotransmitter systems has been suggested as the effect of alcohol withdrawal [18]. In particular, it was hypothesized that an imbalance between neuroexcitatory and neuroinhibitory amino acids may cause many of the symptoms of alcohol withdrawal, at least partially [19]. This hypothesis pointed to Glu and GABA as the main excitatory and inhibitory neurotransmitters, while other neurotransmitters, such as dopamine (DA), serotonin (5-HT), acetylcholine (ACh), and noradrenaline (NA), were assumed to act as regulators. In line with this observation, DA and 5-HT show a deficit in the *nucleus accumbens* during alcohol withdrawal that can rapidly be reversed by alcohol intake [20]. It is becoming better recognized that the various neurotransmitters are not isolated in their functioning, but instead interact with each other through neuronal projections (such as the DA-GABA-Glu pathway) and metabolic pathways. Therefore, substance abuse should be investigated from a systemic point of view.

**Figure 2:**
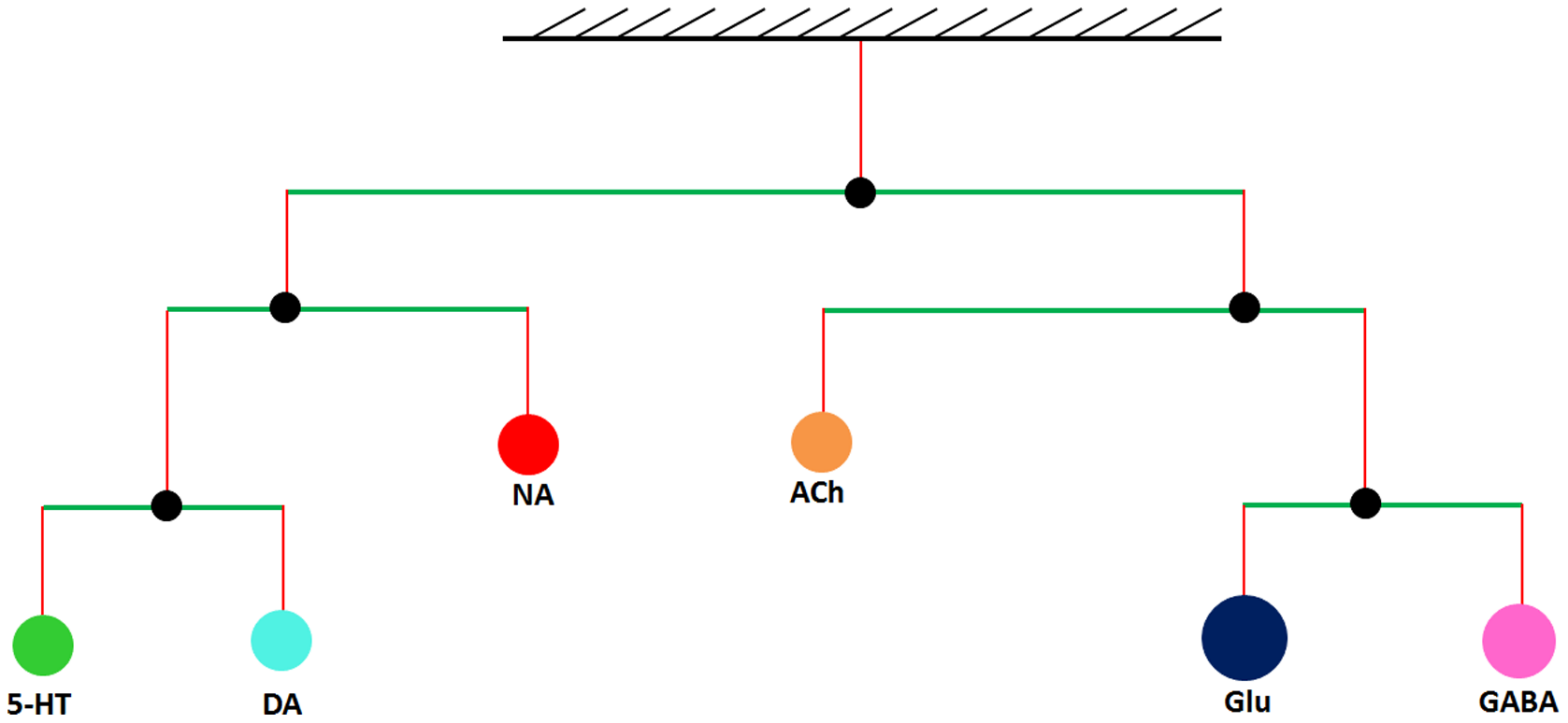
The neurochemical mobile as a system of balanced rods with individual neurotransmitters as weights. The neurochemical mobile represents a hierarchical functional organization of neurotransmitters, along with their relative functional contributions and dynamic imbalances in human brain. The mobile represents synergisms or antagonisms between neurotransmitter systems at the physiological, behavioral, and pathological level, and can be tailored for a specific disease. For illustration, we use a weighting scheme, where the areas of circles are proportional to the relative signal intensities of neurotransmitters 5-HT (100 units), DA (100 units), NA (100 units), ACh (100 units), Glu (200 units), and GABA (150 units). The lengths of the arms of each rod are thus different and reflect corresponding relative signal intensities.

Neurotransmitters not only communicate with each other through a “neurochemical interaction matrix” (Fig. 1), but there is also a delicate dynamic balance between these neurotransmitters from a functional point of view. Disruption of this balance can affect brain function, which in consequence may induce mental disorders. The concept of health and disease as being related to balances and imbalances is at least 2,500 years old, as Hippocrates used the metaphor of the “balance of juices” to describe health status. Inspired by this idea, Fritze suggested studying brain health in terms of balances between neurotransmitters [21]. Subsequently, one of us (FT) constructed a “neurochemical mobile” that represents in a consistent manner different clinical observations of symptoms and of actions of psychopharmacological agents. In this model, different pairs of neurotransmitters form a set of hierarchical balances [22]. The mobile, in its simplest form, includes the neurotransmitters DA, 5-HT, NA on one side, and ACh, Glu, and GABA on the other (Fig. 2). These transmitters, from a functional point of view, mainly act in a more or less opposing mode with each other.

Each component of the neurochemical mobile represents the “relative functional weight” of a neurotransmitter, a term which summarily captures its physiological or behavioral importance and will be discussed later. The functional weight profile determines how the neurotransmitter system is balanced during health or off balance if it is perturbed by substance abuse.

In this study, we explore a combined model of the neurochemical mobile and the neurotransmitter interaction matrix. We are primarily interested in the interactions that correspond to clinical syndromes based on experience and on data of global drug action, regardless of the anatomical distribution of neurotransmitters in the human brain. In particular, we use this modeling platform to simulate alcohol abuse, adaptation, and withdrawal, and analyze the consequences of different schemes in comparison with clinical and experimental experience regarding the balance of neurotransmitters. We also use the model simulations to screen for potential treatments of the symptoms of alcohol withdrawal.

The model proposed here is heuristic and may be categorized as mesoscopic, as its complexity and function fall between those of detailed microscopic (molecular) and high-level macroscopic (physiological and behavioral) models [23]. A mesoscopic model can serve as an important conceptual and cognitive tool, as it rigorously formalizes the logic of argumentation within a complex system, which in a detailed mathematical representation would vastly exceed our intuition [24], [25]. Here, the mesoscopic model forms a critical functional link between lower and higher levels of brain function and provides a conceptual and technical strategy for merging models that characterize specific functional aspects at multiple levels.

## Materials and Methods

The human brain responds differently to the duration and disruption of alcohol intake. Short-term exposure tends to inhibit some brain functions, whereas chronic consumption causes the brain to compensate toward normalcy. When chronic alcohol consumption is disrupted, the direct effects of alcohol disappear, but the compensatory changes are still active, thereby causing symptoms of withdrawal. The model simulations address these mechanisms and processes. They also allow us to explore potential treatments of alcohol withdrawal.

Our modeling framework includes two modules: The first is the neurochemical interaction matrix (Fig. 1) that characterizes interactions between neurotransmitters and their regulations [26]. This model has previously been tested with respect to stability and parameter sensitivities [26]. The second module contains a neurochemical mobile system (Fig. 2) that describes hierarchical functional balances between pairs of neurotransmitters with respect to health and disease [21], [27]. The neurochemical interaction matrix is formulated as a dynamic model with six neurotransmitters, namely DA, ACh, 5-HT, Glu, NA, and GABA. These six neurotransmitters correspond to signals that can regulate each other’s activity. In our model, the interaction matrix is a fully connected network with bidirectional signals (Fig. 1; *cf*. [26] for details). Neurotransmitter concentrations, which may be used to represent signal strengths regulating each other, are available in the literature [28]–[39].

The neurochemical mobile system describes hierarchical functional balances between pairs of neurotransmitters and emulates the idea of a hierarchical “balance scale” (Fig. 2). Each neurotransmitter has a weight representing its relative functional importance, and under normal homeostasis, corresponding to health, all rods are balanced.

The implementation of the neurochemical interaction matrix and the neurochemical mobile system under the categorized weighting scheme is illustrated in Table 1. The implementation presents the ODEs for the neurochemical interaction matrix with specified initial values and the mobile configuration (defined by the lever length profile) for the neurochemical mobile system. As a minimally biased format for the equations, we follow the tenets of Biochemical Systems Theory [40]–[43]. The information in Table 1 should be sufficient for simulations with the model; nonetheless, Matlab code for the system is available as *Supplement S2*.

**Table 1:** Implementation of the neurochemical interaction matrix and the neurochemical mobile system^%^.

Entity	Implementation
Equations^$^	DA' = ACh^0.5^ · HT^0.2^ · Glu^0.5^ · NA^0.5^ · GABA^−0.3^ – 7.901101 · DA
	ACh' = DA^−0.3^ · HT^−0.3^ · Glu^0.5^ · NA^−0.3^ · GABA^−0.3^ – 0.0004985258 · Ach
	HT' = DA^−0.2^ · ACh^0.5^ · Glu^0.5^ · NA^−0.3^ · GABA^−0.3^ – 0.03145485 · HT
	Glu' = DA^−0.3^ · ACh^0.5^ · HT^0.5^ · NA^0.5^ · GABA^−0.3^ – 0.2793461 · Glu
	NA' = DA^0.5^ · ACh^0.5^ · HT^0.5^ · Glu^0.5^ · GABA^−0.3^ – 31.45485 · NA
	GABA' = DA^−0.3^ · ACh^0.5^ · HT^−0.3^ · Glu^0.5^ · NA^−0.3^ – 0.01494252 · GABA
Initial values^#^	HT = 100; DA = 100; NA = 100;
	Glu = 200; GABA = 150; ACh = 100;
Mobile configuration (Lever length)^&^	Lever_HT = 1; Lever_DA = 1; Lever_NA = 2;
	Lever_HT_DA = 1; Lever_HT_DA_NA = 1;
	Lever_Glu = 1; Lever_GABA = 1.3333; Lever_ACh = 3.5;
	Lever_Glu_GABA = 1; Lever_ACh_Glu_GABA = 0.6667;

%: This implementation of the system uses the categorized weighting scheme, which assumes that the contributions of the different neurotransmitters to alcohol dependence are of comparable impact. Specifically, the concentrations are categorized into classes and transformed into low (100), medium (150), and high (200) values. A Matlab file of the system is presented in *Supplement S2*.

$: Apostrophes on the left-hand sides of the equations indicate derivatives with respect to time.

#: Relative signal intensities of neurotransmitters.

&: Lever lengths in the mobile for the various neurotransmitters. Please see the Figure 2 for the corresponding positions of levers within the mobile.

To model the acute alcohol usage, we increase GABA synthesis and decrease Glu synthesis in the neurochemical interaction matrix. These direct effects trigger compensatory mechanisms during chronic usage that are implemented as an increase in GABA degradation and a decrease in Glu degradation in the neurochemical interaction matrix. Again, these direct effects trigger secondary responses by the system. During alcohol withdrawal, the alcohol effects are removed, but the compensatory mechanisms of the brain are taken into account.

For the functional weights within the mobile, we assume that the contributions of the different neurotransmitters to alcohol dependence are of comparable impact, even though their actual concentrations in the brain differ quite considerably. The rationale is that neurotransmitters with normally small concentrations are correspondingly more potent. Specifically, we transform the concentrations into low, medium and high magnitudes and assign 100, 150, and 200 as relative signal intensities of neurotransmitters, respectively. These relative signal intensities are used to compute the profile of lever lengths in the mobile during health. In addition, they are used as neurotransmitter “signal intensities” in the neurochemical interaction matrix, which characterizes signal interactions between neurotransmitters.

As an alternative, the functional weights of neurotransmitters could directly be equated with their concentrations. This option and its ramifications are discussed in *Supplement S1*. The length of each lever arm relative to its counterpart is computed under the assumption of perfect balance, which means that the two lever arms of a balance scale in our mobile can have different lengths. To show an imbalance within the mobile properly, and to prevent a rotation angle from reaching ±90°, we add a resistant force at the end of each lever arm, which is proportional to the vertical displacement of the end of a lever arm from its corresponding balanced position. In this manner, we can properly compute and visualize dynamic imbalances within the mobile. To describe an imbalanced state unambiguously, we define counterclockwise and downward as positive directions for rotation and force, respectively. One should note that the balances within the mobile are governed by both the length profile of the arms (determined by the profile of functional weights) and the intensities of neurotransmitter signals (determined by the neurochemical interaction matrix) in a time-dependent manner. Thus, the mobile is dynamic. The computation of the appropriate rotation angle of a rod in the mobile is shown in Figure S3 in Supplement S1.

## Results

**Figure 3:**
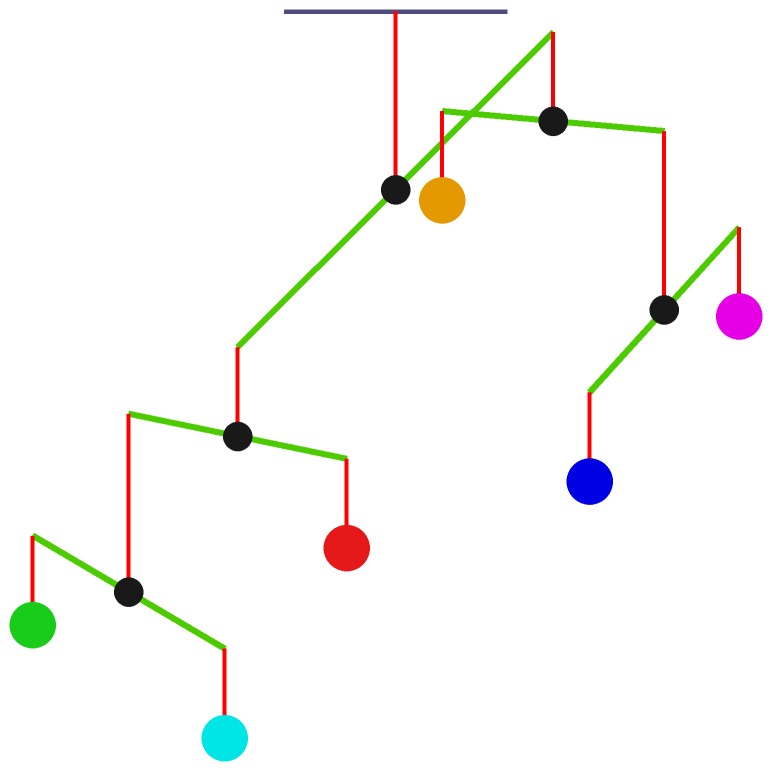
In response to alcohol withdrawal, the mobile system exhibits significant imbalance. Circles from left to right: 5-HT, DA, NA, ACh, Glu, and GABA. For visibility, the lengths of arms in the mobile are adjusted as described, and neurotransmitter concentrations are transformed into comparable relative signal intensities, so that all neurotransmitters have similar functional weights.

**Figure 4:**
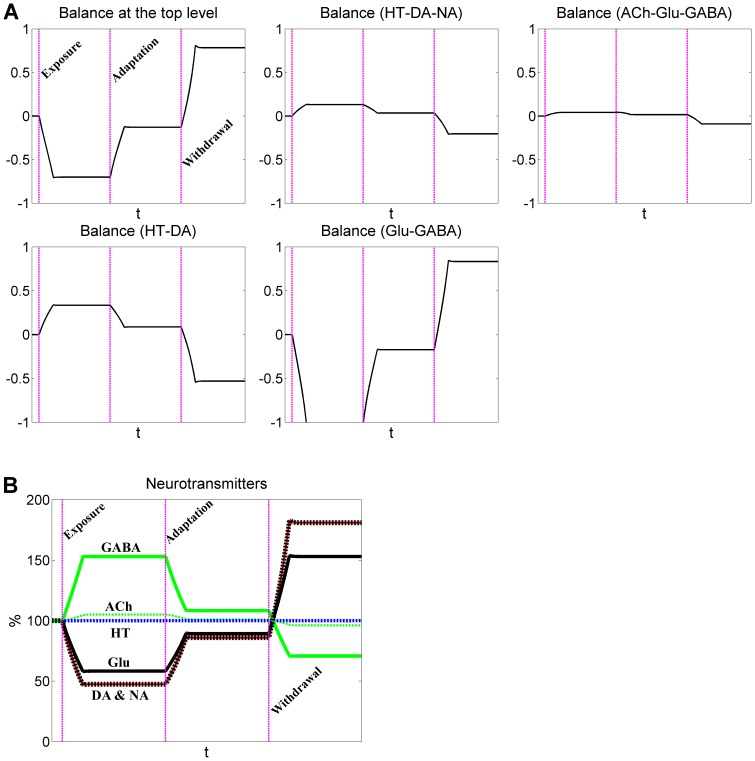
Dynamics of neurotransmitters and rod balances in response to acute alcohol exposure, adaptation, and withdrawal. Neurotransmitters have similar functional contributions in the mobile system. The three vertical dashed lines represent, from left to right, the beginning of acute alcohol exposure, adaptation, and withdrawal, respectively. A: Dynamic imbalances associated with five rods are represented by rotation angles relative to the horizontal position. B: Dynamics of neurotransmitters. Note that the dynamic responses of DA and NA overlap.

The overall state of the mobile is greatly disturbed by alcohol. Indeed, all rods exhibit imbalances, some of which are quite significant (Fig. 3). As a direct consequence of acute exposure to alcohol, the rod connecting Glu and GABA tilts toward GABA. If the acute exposure turns into repeated abuse, the body adapts, but full compensation cannot be achieved, and the rod eventually becomes less slanted than before but still not level. During withdrawal, the mobile tilts back toward the excitatory neurotransmitter Glu (Fig. 4).

In more detail, the top rod, which balances DA, NA, and 5-HT on the left, and Glu, GABA and ACh on the right, tilts toward the right under acute exposure. For repeated abuse, it moves back toward normalcy, thereby reflecting adaptive compensation, which however is not perfect. During withdrawal, the rod tilts back toward DA, NA, and 5-HT. Another significant imbalance is seen in the rod connecting 5-HT and DA, which tilts toward DA during withdrawal. One interesting phenomenon in this context is that the neurochemical interaction matrices exhibit only subtle differences in dynamic responses for the two alternative definitions of functional weights (Fig. 4B and Figure S1B in Supplement S1), although the two mobiles show distinctly different imbalance profiles (Fig. 3 and Figure S2 in Supplement S1).

The results raise the question of whether a quasi-balance could be regained with proper treatment. Several medications are currently available to treat the symptoms of alcohol withdrawal. Table 2 shows some of these, along with their pharmacological mechanisms. Most of them act on the GABA receptor and enhance GABA signals. Others affect the Glu or NA systems.

**Table 2:** Partial list of medications for symptoms of alcohol withdrawal.

Generic name (brand name)	Pharmacological mechanism
Diazepam (Valium or Diastat)	Binds to the GABA_A_ receptor and results in enhanced GABA effects
Chlordiazepoxide (Librium or Angirex)	Modifies GABA_A_ receptor and increases its overall conductance
Clonidine (Kapvay or Nexiclon)	Binds to α_2_ receptors and inhibits the release of NA

**Figure 5:**
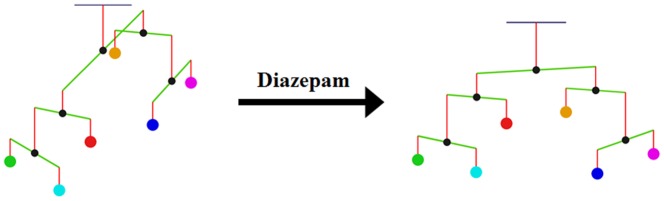
Diazepam reduces imbalances caused by alcohol withdrawal. The mobile confirms that diazepam reduces imbalances during alcohol withdrawal. Circles from left to right: 5-HT, DA, NA, ACh, Glu, and GABA. For better visibility, the lengths of arms in the mobile are adjusted and do not reflect functional contributions of neurotransmitters.

**Figure 6:**
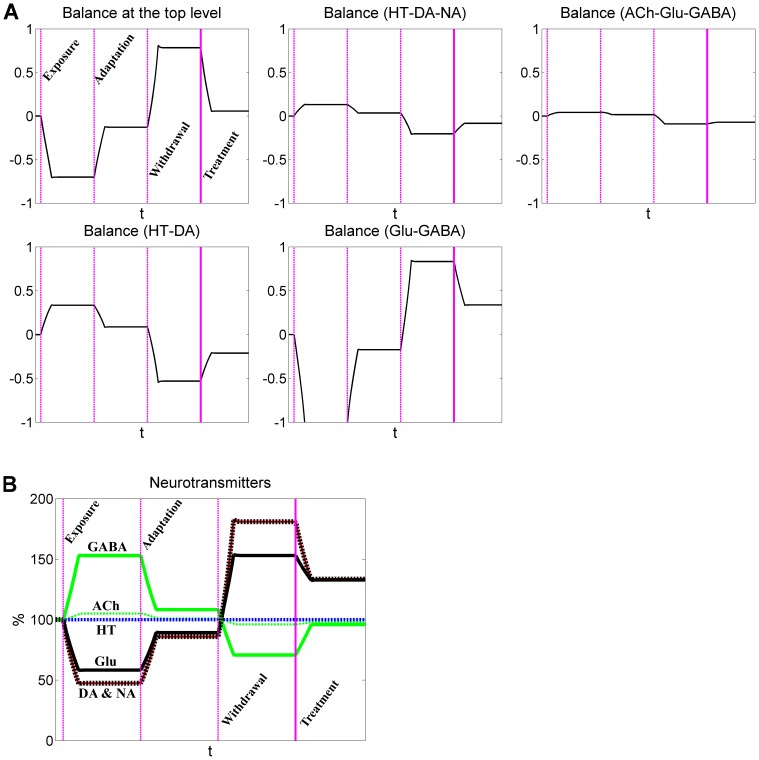
Diazepam partly restores the balance between excitatory and inhibitory neurotransmitters. Neurotransmitters have similar functional contributions in the mobile system. The three dashed vertical lines represent, from left to right, the beginning of acute alcohol exposure, adaptation, and withdrawal, respectively. The solid vertical line represents the application of diazepam. A: Dynamic imbalances associated with five rods are represented by rotation angles relative to the horizontal position. B: Dynamics of neurotransmitters. Note that the dynamic responses of DA and NA overlap.

As a prominent example, consider diazepam, which activates the GABA_A_ receptor. It is not difficult to simulate this effect, and the model results directly confirm that diazepam clearly reduces the overall imbalance of the mobile system during alcohol withdrawal (Fig. 5). Even though diazepam only targets the GABA signal directly, all other neurotransmitters are indirectly affected through the neurochemical interaction matrix, as it is shown in Figure 6. For example, the excitatory Glu is reduced and the inhibitory GABA is elevated. These changes help to alleviate perturbations induced by alcohol withdrawal.

In addition to medications acting on the GABA signal, our simulations confirm the beneficial effects of pharmacological actions on the NA signal and suggest a potential target of the ACh system (data not shown). With regard to actions on the NA signal, one medication, Clonidine (Kapvay or Nexiclon), is already in use. The effect on the ACh system is revisited in the discussion section.

## Conclusions and Discussion

Over many decades, biologists and clinicians have been accumulating large amounts of data and information on drug abuse and withdrawal, mainly from targeted animal studies, clinical trials, and observations on individual patients. The assemblage of knowledge resulting from these efforts is indeed impressive and very valuable, but also poses the challenge of integrating the diverse and heterogeneous pieces of information into a common functional entity that can be analyzed, interrogated, and used to formulate new hypotheses. Such an integrative investigation of a phenomenon like drug dependency requires a systemic point of view, which in turn mandates the use of computational means of data management and the development of dynamic mathematical models. These computational tools are well suited for the purpose because they are able to accommodate and merge diverse data sets that were obtained from genetic, biochemical, pharmacological, and clinical investigations.

In this study, we presented a systemic model of neurotransmitter interactions to address questions of alcohol abuse and withdrawal. The model is heuristic and intuitive and, while conceptually simple, can simulate the effects of genetic mutations, biochemical interventions, and pharmacological actions. For example, our simulations demonstrate the actions of the drug diazepam for alcohol withdrawal symptoms quite well. By integrating various pieces of information, the dynamic framework allowed us to search for explanations of the details of alcohol dependency and permitted simulations of currently used as well as hypothesized treatment options.

The heuristic model combines a neurotransmitter interaction matrix with the concept of a neurochemical mobile. It is incomparably simpler than complicated dynamic models of neurotransmitters, such as our detailed nonlinear ordinary differential equation models of the dopamine dynamics in neurons [44]–[46], but nevertheless capable of connecting molecular events to the physiological or behavioral deviations from normalcy that are observed during alcohol abuse and withdrawal. The model is mesoscopic and connects otherwise isolated levels of functioning, namely the dynamics of molecular events at the neurotransmitter level and the responses of alcohol usage and withdrawal at the high level of physiology and behavior [23]–[25]. In doing so, the combined matrix-interaction-mobile model opens new avenues of integrating formerly isolated pieces of information about alcohol dependence that can be attributed to different neurotransmitter systems.

As an example, perturbations may be introduced into a detailed dynamic model of a neurotransmitter at the molecular level, and the perturbed neurotransmitter level can then be fed into the neurotransmitter matrix, which then reveals changes in neurotransmitter balance and consequent mobile imbalance. With this combination, the effects of the perturbation at the physiological or behavioral level are quantified through the mobile model. In a different application, one may use the mobile model and the neurotransmitter interaction matrix to explore different scenarios of neurotransmitter alterations that can induce a particular phenomenon of interest. In this case, the possible causes of the neurotransmitter alterations may be assessed with relevant dynamic models, which in turn may also suggest potential interventions.

The model suggests that, following alcohol exposure, the main inhibitory neurotransmitter GABA is enhanced, while the main excitatory neurotransmitter Glu is suppressed. In response to withdrawal, these changes are opposite to those during the acute exposure phase: Glu is elevated, while GABA is reduced. These simulation results are consistent with experimental observations [13]–[17], [47]. In addition to Glu and GABA, the model identifies DA as being over-active during alcohol withdrawal, a result that is supported by several experiments [48]–[51], although not by all [20]. Consistent with experimental data, NA is also increased following alcohol withdrawal in our simulations (Fig. 4 and Figure S1 in Supplement S1) [50]–[52]. Our model does not show significant changes in ACh and 5-HT in response to alcohol abuse and withdrawal. This result is partially supported by a study of the effects of neuropharmacological agents on seizures during withdrawal [53].

The model demonstrates that the drug diazepam for symptoms of alcohol withdrawal effectively reduces the imbalances between neurotransmitters. It also suggests the importance of the NA signal, which is targeted by the prescription drug Clonidine (Kapvay or Nexiclon).

Interestingly, the model suggests the ACh signal as a novel target for treatment of symptoms associated with alcohol withdrawal. It appears that there is currently no drug available that acts on the ACh signal for the treatment of alcohol withdrawal. If the model prediction points into the right direction, this site could be a potential drug target for treating symptoms of alcohol withdrawal and therefore be of pharmaceutical interest. By contrast, our results suggest that DA is not likely an effective drug target, because perturbing DA alleviates the excitatory Glu but exacerbates the inhibitory GABA, or *vice versa* (data not shown). A similar effect is observed if haloperidol is administered as the sole medication to treat a *delirium tremens*. To be effective, a GABA agonist is needed in addition to haloperidol.

Beyond alcohol dependence, the constellation of counteracting transmission systems in the mobile model matches clinical experience with other neurological problems, such as the features of depression, the action of antidepressants, and some side effects of current treatment options. In depression, one encounters low levels of DA, NA and 5-HT transmission, while schizophrenia is associated with high levels of transmission in DA and 5-HT. The mobile permits the investigation of these neurotransmitter profiles, as well as medication strategies for the different states of allostasis.

Neurotransmitter concentrations *in situ* differ greatly; *e.g.*, by three or four orders of magnitude between Glu and DA in the synaptic cleft. This large difference could imply that neurotransmitters with high concentrations, such as Glu and GABA, are the primary players in brain physiology and behavior. Our study, however, suggests that other neurotransmitters seem to be of comparable importance, at least in the case of alcoholism, and that the relative magnitudes to baseline levels, rather than the absolute concentrations, are more appropriate for capturing their physiological and behavioral impact.

The matrix model leaves some questions unresolved. One of them is: What are the primary targets of alcohol, and which neurotransmitters are not directly targeted, although they are greatly affected indirectly through interactions among the neurotransmitters? From experimental observations, both Glu and GABA are frequently and consistently reported to be targeted by alcohol. However, experimental data on DA activity under alcohol withdrawal are contradictory. Since Glu and GABA are mostly reported as alcohol targets, we considered them as the primary targets in our model, but it could as well be the case that NA and DA are first responders. In the future, it might be interesting to investigate systemically whether there could be different scenarios of primary alcohol targets that are consistent with clinical data and provide explanations for so-far contradictory observations.

In this study, we treated each type of neurotransmitters as a single node in the neurotransmitter interaction matrix. However, each type of neurotransmitters has its own subtypes of receptors, which can have opposite actions between two subtypes. In future work, one might account for these subtypes of neurotransmitter receptors and treat them as different nodes in a much larger interaction matrix based on their actions (*e.g.*, grouped into different categories).

## Supporting Information

Supplement S1 **Supporting text and figures. Figure S1.** Dynamics of neurotransmitters and balances in response to acute alcohol exposure, adaptation, and withdrawal. The neurotransmitter concentrations are directly used as functional weights. The three dashed vertical lines represent, from left to right, the beginning of acute alcohol exposure, adaptation, and withdrawal, respectively. A: Dynamic imbalances associated with five rods are represented by rotation angles relative to the horizontal position. B: Dynamics of neurotransmitters. Note that the dynamic responses of DA and NA overlap. **Figure S2.** In response to alcohol withdrawal, the mobile system does not exhibit significant imbalances. Here, the neurotransmitter concentrations are directly used as functional weights. Circles from left to right: 5-HT, DA, NA, ACh, Glu, and GABA. For better visualization, the lengths of the arms in the mobile are adjusted and do not reflect functional contributions of neurotransmitters. **Figure S3.** Computation of a rotation angle of a scale in the mobile system. The scales in the mobile can have different lengths of lever arms. *A* and *B* are the signal intensities of two neurotransmitters in this study. *θ* is the rotation angle, while *r_a_* and *r_b_* are the lengths of lever arms. F_a_ and F_b_ are resistant forces, while *k* is the coefficient of resistant force.
(DOCX)

Supplement S2 (M)
