# Supplementary material for: A Heuristic Model of Alcohol Dependence
Source: PLoS One. 2014 Mar 21;9(3):e92221. doi: 10.1371/journal.pone.0092221 (PMC3962390; doi:10.1371/journal.pone.0092221)
Supplement: Supplement S1 — Supporting text and figures. Figure S1. Dynamics of neurotransmitters and balances in response to acute alcohol exposure, adaptation, and withdrawal. The neurotransmitter concentrations are directly used as functional weights. The three dashed vertical lines represent, from left to right, the beginning of acute alcohol exposure, adaptation, and withdrawal, respectively. A: Dynamic imbalances associated with five rods are represented by rotation angles relative to the horizontal position. B: Dynamics of neurotransmitters. Note that the dynamic responses of DA and NA overlap. Figure S2. In response to alcohol withdrawal, the mobile system does not exhibit significant imbalances. Here, the neurotransmitter concentrations are directly used as functional weights. Circles from left to right: 5-HT, DA, NA, ACh, Glu, and GABA. For better visualization, the lengths of the arms in the mobile are adjusted and do not reflect functional contributions of neurotransmitters. Figure S3. Computation of a rotation angle of a scale in the mobile system. The scales in the mobile can have different lengths of lever arms. A and B are the signal intensities of two neurotransmitters in this study. θ is the rotation angle, while ra and rb are the lengths of lever arms. Fa and Fb are resistant forces, while k is the coefficient of resistant force. (DOCX) [file pone.0092221.s001.docx]

**Supplement 1: A Heuristic Model of Alcohol Dependence**

**Zhen Qi, Felix Tretter, Eberhard O. Voit**

**Definition of functional weights**

Conceptually, the functional weights determine how the neurotransmitter system is balanced during health or off balance if it is perturbed by external factors, such as substance abuse. So far, no targeted study has shed light on the quantification of relative functional weights, and a firm definition of the term “functional weight” has not yet been established. One challenge for the quantification of relative functional weights is that it is not known whether each disease phenomenon (*e.g.*, alcohol withdrawal) may require its own specific quantification.

The most intuitive definition is directly based on the concentration profile of the involved neurotransmitters. These concentrations by and large determine the intensity of input signals, because neurotransmitters bind to their receptors in the process of transducing information. However, this direct assessment might be somewhat too simplistic, and therefore requires caution. For instance, different neurotransmitters have very different receptor redundancies and signal transduction mechanisms, and neurotransmitter concentrations may therefore not properly represent signal intensity after receptor binding and signal transduction pathway activation. Also, the concentrations of neurotransmitters in the synaptic cleft differ greatly: Dopamine is found at the *nM* scale [[29](#_ENREF_29)], while the concentration of glutamate, at the *µM* scale, is several orders of magnitude higher [[30](#_ENREF_30)]. If the concentrations in the synaptic cleft were used directly to represent relative functional contributions, one molecule of glutamate would be functionally equivalent to thousands of dopamine molecules. Such a stark difference in effect may not always be an appropriate representation of functional contributions.

Instead of using concentrations directly, we made, in the body of the paper, the assumption that the neurotransmitters have comparable signal intensities even if their concentrations differ substantially. This argument suggests that all neurotransmitters in the mobile should be assigned functional weights within the same order of magnitude. Specifically, the effects of one molecule of two different neurotransmitters are not necessarily functionally equal, but the concentrations are categorized into classes that are coded as low, medium, and high values of the same order of magnitude. As these questions fall into uncharted territory, we tested different weighting schemes for the case of alcohol dependence. The results suggest that the assumed weighting scheme of similar functional contribution among neurotransmitters appears to capture the physiological and behavioral impact of these neurotransmitters appropriately.

When the neurotransmitter concentrations are directly used as functional weights, different neurotransmitters show different dynamic responses to alcohol intoxication (Fig. S1). Following acute alcohol exposure, the main inhibitory neurotransmitter GABA is enhanced, while the main excitatory neurotransmitter Glu and the two regulatory amino acids DA and NA are suppressed. In response to chronic exposure, the brain evokes adaptation mechanisms, causing all neurotransmitters to approach levels close to normalcy. Finally, during withdrawal, the excitatory neurotransmitter Glu becomes elevated in comparison to its base level, while the inhibitory neurotransmitter GABA is reduced. Thus, the brain enters a hyper-activity state, leading to symptoms of withdrawal. However, the mobile with concentration-based weights does not generate significant imbalances under alcohol exposure. Among the five rods in the mobile system, only the rod connecting Glu and GABA shows a significant imbalance, while all other rods are very close to their balanced states (Fig. S2), which is not consistent with clinical experience.

By contrast, the categorized weighting scheme causes the mobile to produce significant imbalances in response to alcohol abuse and withdrawal, and these imbalances are in line with clinical observations. This selected weighting scheme assumes similar functional contributions of different neurotransmitters even if their concentrations differ by several orders of magnitude. The consequence of this scheme is seen in greatly disturbed hierarchical balances, and this degree of disturbance seems closer to clinical observations than other weighting schemes, such as the concentration-based scheme (Fig. 3 & S2).

The reason that this scheme could be more appropriate might be that neurotransmitter binding to corresponding receptors, and the subsequent transduction of signals, critically depends on receptor abundance, binding activity, and other factors that strongly differ among neurotransmitter systems. Thus, there is a correspondence between vastly different concentrations and signal receiving entities.

Caution while interpreting these results is necessary, nevertheless, because the validity of our conclusions is dependent on the premise that the mobile represents the functional relationships between neurotransmitters at the level of physiological and behavioral function for alcohol abuse and withdrawal, and this premise still requires experimental tests.


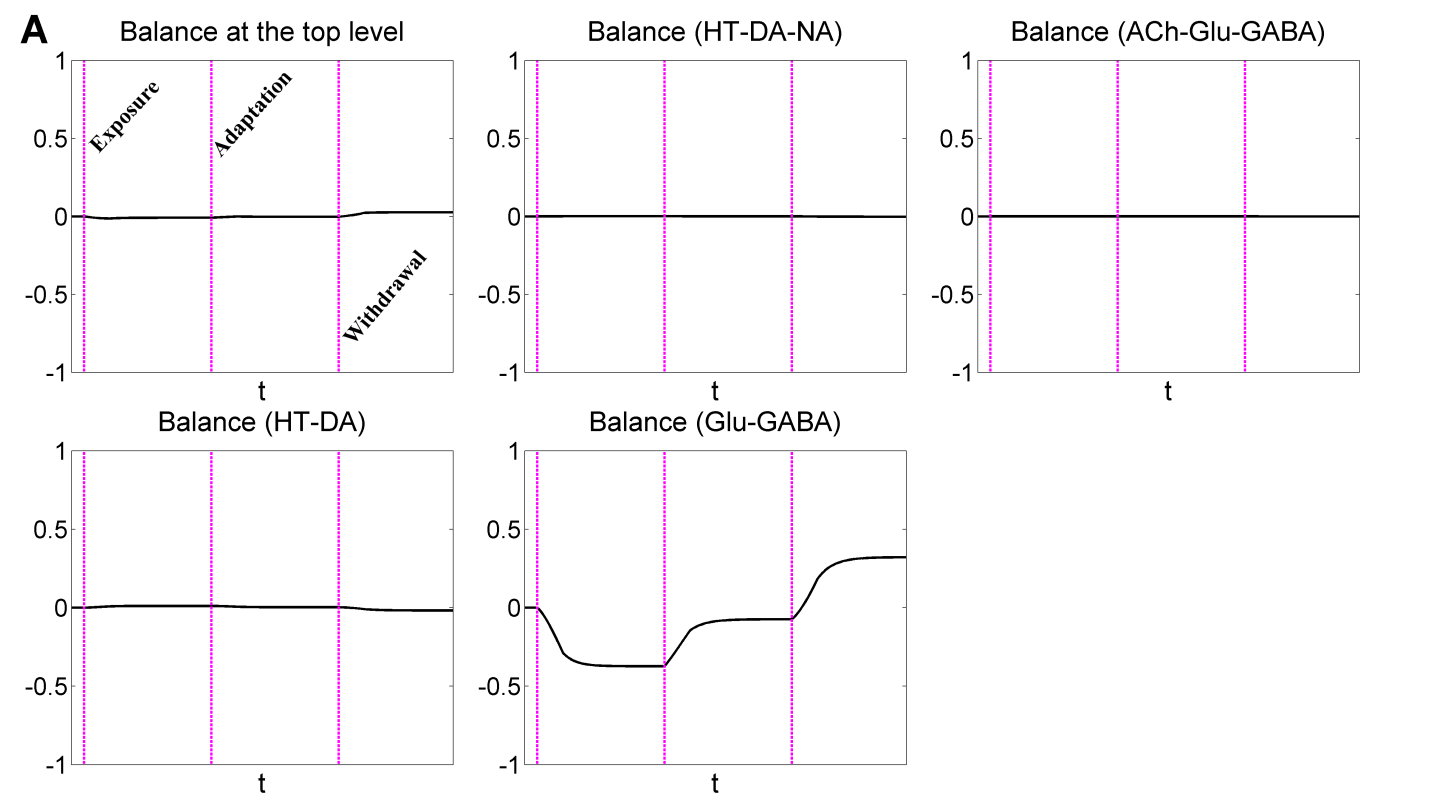


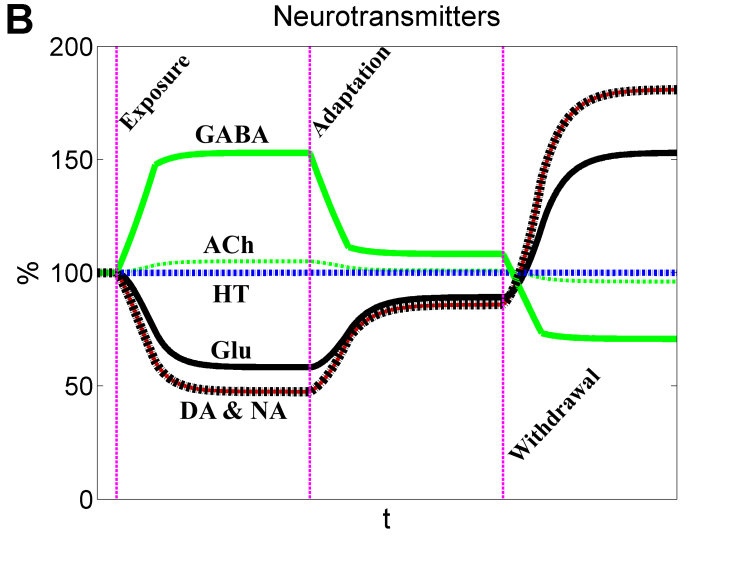


**Figure S1: Dynamics of neurotransmitters and balances in response to acute alcohol exposure, adaptation, and withdrawal.** The neurotransmitter concentrations are directly used as functional weights. The three dashed vertical lines represent, from left to right, the beginning of acute alcohol exposure, adaptation, and withdrawal, respectively.

A: Dynamic imbalances associated with five rods are represented by rotation angles relative to the horizontal position.

B: Dynamics of neurotransmitters. Note that the dynamic responses of DA and NA overlap.

In contrast to the mobile, the dynamics of the neurochemical interaction matrix is very similar between the two different weighting schemes. This similarity in dynamics reflects that the overall effect of a perturbation depends much more on the structure of the neurotransmitter interaction matrix rather than on specific values representing the neurotransmitters. By contrast, the balance and imbalance of the mobile strongly depend on both the model structure and numerical values.

**
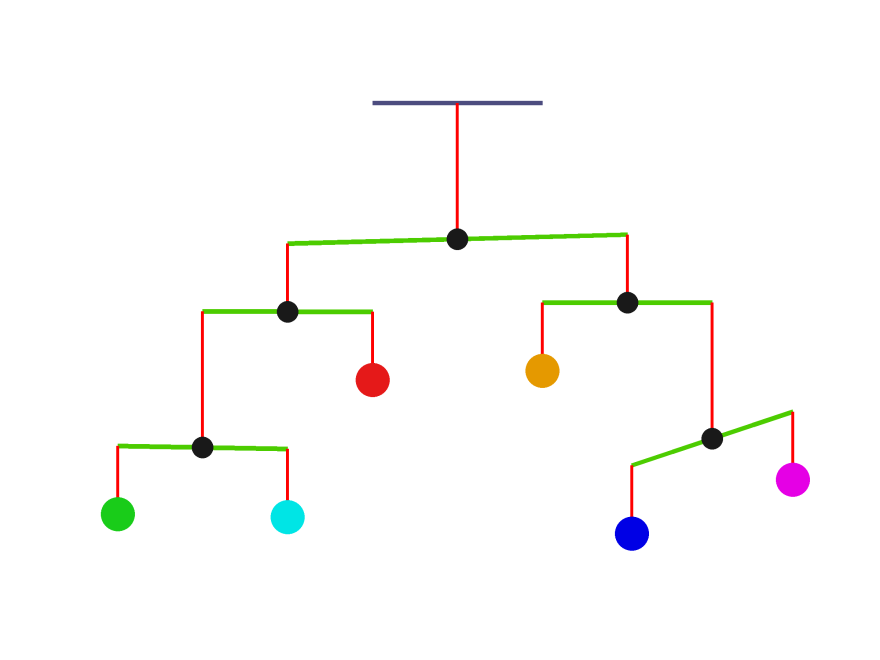
**

**Figure S2: In response to alcohol withdrawal, the mobile system does not exhibit significant imbalances.** Here, the neurotransmitter concentrations are directly used as functional weights. Circles from left to right: 5-HT, DA, NA, ACh, Glu, and GABA. For better visualization, the lengths of the arms in the mobile are adjusted and do not reflect functional contributions of neurotransmitters.

**Computation of the rotation angle of a rod in the mobile system**

The lever arms of a scale in the mobile can have different lengths. To show an imbalance within the mobile properly, and to prevent a rotation angle from approaching ±90° under the smallest of perturbations, we add a resistant force at the end of each lever arm, which is proportional to the vertical displacement of the end of a lever arm from its corresponding balanced position. Since the lever arms of a scale can have different lengths, the resistant forces at two sides of a scale can also be different.

Let’s consider an example of the computation of a rotation angle of a rod in the mobile system (Fig. S3). We define counterclockwise and downward as positive directions for rotation and force, respectively. When the scale is off balance, it rotates and the resistant forces change accordingly. Finally, the scale reaches a new balanced state that is represented by the rotation angle. At the new balanced state, the sum of torques on the scale equals zero, and this fact is used to compute to the rotation angle as shown by the following equations:

$(A-k\sin\theta\cdot r_{a})\cdot r_{a}\cos\theta=(B+k\sin\theta\cdot r_{b})\cdot r_{b}\cos\theta$ (1)

$\sin\theta=\frac{Ar_{a}-Br_{b}}{(r_{a}^{2}+r_{b}^{2})\cdot k}$ (2)

Here, *A* and *B* are the signal intensities of two neurotransmitters at the ends of the same rod. *θ* is the rotation angle, while *r_a_* and *r_b_* are the lengths of lever arms. The parameter *k* is the coefficient of resistant force. These equations apply to clockwise and counter-clockwise rotation. The sum of two signal intensities and two resistant forces is the following, which is needed for the computation of rotation angles of other scales in the hierarchical mobile.

$\sum F=A+B+k\sin\theta\cdot(r_{b}-r_{a})$ (3)





**Figure S3: Computation of a rotation angle of a scale in the mobile system.** The scales in the mobile can have different lengths of lever arms. *A* and *B* are the signal intensities of two neurotransmitters in this study. *θ* is the rotation angle, while *r_a_* and *r_b_* are the lengths of lever arms. F_a_ and F_b_ are resistant forces, while *k* is the coefficient of resistant force.
